# Supplementary material for: Multiobjective Design of Growth Media with Genome-Scale Metabolic Models and Bayesian Optimization
Source: Comput Struct Biotechnol J. 2026 May 7;35(1):0072. doi: 10.34133/csbj.0072 (PMC13150070; doi:10.34133/csbj.0072)
Supplement: Supplementary 1 — Figs. S1 to S6 Tables S1 to S3 [file csbj.0072.f1.pdf]

Supplementary Figures and Tables

Multiobjective design of growth media with genome-scale metabolic models and Bayesian optimization

Nicola Hallmann[1], Catalina Guerra-Cornejo[2], Karl Burgess[2], Charlotte Merzbacher[3,†], Diego A. Oyarzún[2,3,†]

[1]Department of Biosystems Science and Engineering, ETH Zurich, Switzerland

[2]School of Biological Sciences, University of Edinburgh, UK

[3]School of Informatics, University of Edinburgh, UK

[†]Corresponding authors: cjmerzbacher@gmail.com; d.oyarzun@ed.ac.uk

Table S1: **Medium components for case studies in *Escherichia coli*.** Unless otherwise specified, all numerical values are given as fluxes in mmol $gDW^{-1}h^{-1}$. The concentrations were converted to fluxes by applying the scaling factor discussed in Section 4.3. Prices for components with a fixed concentration (trace metals usually provided by a 1000x Trace Metals Mixture (1000x TMM)) and for components provided by the general infrastructure (oxygen) were set to zero. All other prices were taken from the Sigma Aldrich UK website in October 2024. The medium for iML1515 contains all listed components except for the amino acids. The medium for the iJO1366-antiEpEX-scFv model contains all listed components.

Medium Component	M9/Optimal Concentration [mM]	Scaled M9/Optimal Concentration	Lower Bound	Upper Bound	Cost [£/mol]	Source(s)
Ammonium (NH_4^+)	18.695	9.3475	0	10	10.099587	NH_4Cl
Calcium(II)	0.1	0.05	0	10	18.08223	$CaCl_2 \cdot 2HO$
Chloride	8.656	13.6755	0	20	3.03888	NaCl, NH_4Cl, $CaCl_2 \cdot 2HO$
Cobalt(II)	0.0002	0.0001	0.0001	0.0001	0	1000x TMM
Copper(II)	0.002	0.001	0.001	0.001	0	1000x TMM
Glucose	20	10	1	10	7.7647236	Glucose
Iron(II)	0.2	0.1	0.1	0.1	0	1000x TMM
Manganese(II)	0.002	0.001	0.001	0.001	0	1000x TMM
Magnesium(II)	2.0	1	0	10	19.1022	$MgSO_4 \cdot 7H_2O$
Molybdenum(II)	0.001	0.0005	0.0005	0.0005	0	1000x TMM
Nickel(II)	0.002	0.001	0.001	0.001	0	1000x TMM
Oxygen (O_2)	40	20	0	20	0	air in incubator
Phosphate (PO_4^{3-})	69.8	34.9	0	50	23.4234	Na_2HPO_4, KH_2PO_4
Potassium	22.04	11.02	0	20	20.82177	KH_2PO_4
Tin(II)	0.002	0.001	0.001	0.001	0	1000x TMM
Sodium	104.076	52.038	0	100	0	Na_2HPO_4, NaCl
Sulfate (SO_4^{2-})	2.0	1	0	10	19.1022	$MgSO_4 \cdot 7H_2O$
L-Arginine	9.5	4.75	0	10	61.1442	L-Arginine
L-Asparagine	6.1	3.05	0	10	93.01248	L-Asparagine
L-Glutamine	9.9	4.95	0	10	80.23086	L-Glutamine

Table S2: **Medium components for case study in *Bacillus subtilis*.**. Unless otherwise specified, all numerical values are given as fluxes in mmol $gDW^{-1}h^{-1}$. The concentrations were converted to fluxes by applying the scaling factor discussed in Section 4.3. The medium for iBsu1103 contains all the components from the M9 medium except for chloride and iron, as only trace metals.

Medium Component	M9/Optimal Concentration [mM]	Scaled M9/Optimal Concentration	Lower Bound	Upper Bound	Source(s)
Ammonium (NH_4^+)	18.695	9.3475	0	10	NH_4Cl
Calcium(II)	0.1	0.05	0	10	$CaCl_2{\cdot}2HO$
Glucose	20	10	1	10	Glucose
Iron(II)	0.2	0.1	0	10	$FeSO_4$
Magnesium(II)	2.0	1	0	10	$MgSO_4{\cdot}\ 7H_2O$
Oxygen (O_2)	40	20	0	20	air in incubator
Phosphate (PO_4^{3-})	69.8	34.9	0	50	Na_2HPO_4, KH_2PO_4
Potassium	22.04	11.02	0	20	KH_2PO_4
Sulfate (SO_4^{2-})	2.0	1	0	10	$MgSO_4{\cdot}\ 7H_2O$
Carbon Dioxide (CO_2)	N/A	0	0	10	produced in fermentation
Protons (H^+)	N/A	0	0	10	produced in fermentation
Water (H_2O)	N/A	N/A	0	100	solvent

Table S3: **Runtime profiling of gsMOBO.** We employed the `cProfile` utility to study the distribution of runtime across the various gsMOBO components, using the GEM for antibody production in *Escherichia coli*. We ran the triple objective optimizations in Figure 3 (growth-production-cost, gpc) and Supplementary Figure S4 (growth-production-cost with amino acids only; gpc_M9_fix), alongside the combinations of double optimizations (Supplementary Figures S1–S3; gc, gp, pc). Shown are the runtimes of the `find-next-candidate` routine as a fraction of total runtime, broken down into the subroutines for optimizing the acquisition function (`optimize-acqf-list`) and training of the Gaussian Process regressor (`fit-gpytorch-mll`). In all cases gsMOBO was called with the same parameters specified in the manuscript; code profiling was done on a Lenovo ThinkPad T14 Gen6 with an AMD Ryzen AI 7 PRO 350 processor (8 cores, 16 threads; base clock 2.0 GHz).

		iJO1366-antiEpEX-scFV				
n_iter		100	100	100	100	100
optimization objective		gc	gp	pc	gpc	gpc_M9_fix
number of objectives		2	2	2	3	3
number of decision variables		13	13	13	13	3
runtime [s]		3810	5436	8094	6064	3246
Function						
`find_next_candidate`	cumtime [s]	3685	5308	7963	5944	3146
	% of runtime	97%	98%	98%	98%	97%
	ncalls	100	100	100	100	100
`optimize_acqf_list`	cumtime [s]	2329	3250	2342	3512	2507
	% of run time	61%	60%	29%	58%	77%
	ncalls	100	100	100	100	100
`fit_gpytorch_mll`	cumtime [s]	1339	2046	5608	2404	621
	% of runtime	35%	38%	69%	40%	19%
	ncalls	300/100	300/100	300/100	300/100	400/100

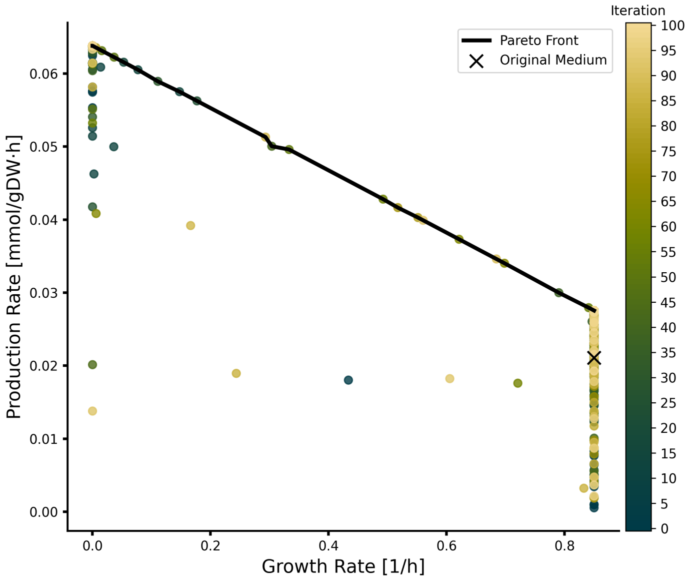

Figure S1: **Double-objective optimization of antibody-producing *E. coli* GEM medium conditions.** The optimization, with the objective to maximize both growth rate and production rate, was run for N=100 iterations, a batch size of 15, and 50 random starting medium compositions.

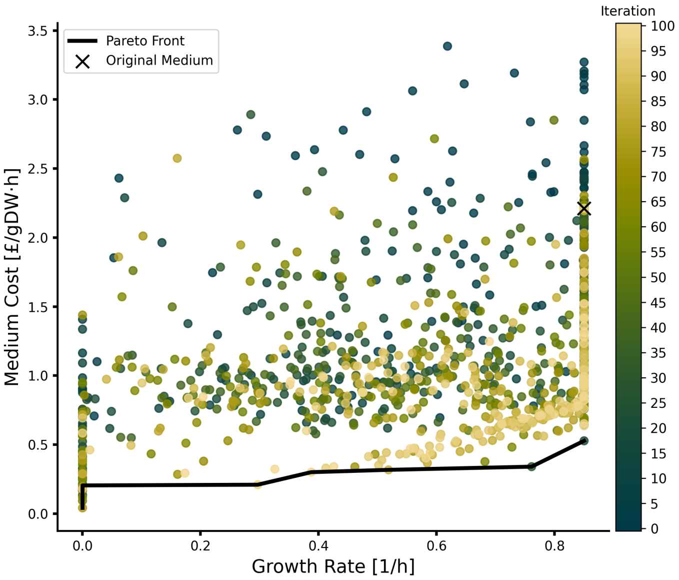

Figure S2: **Double-objective optimization of antibody-producing *E. coli* GEM medium conditions.** The optimization, with the objective to maximize the growth rate while minimizing the cost, was run for N=100 iterations, a batch size of 15, and 50 random starting medium compositions. For readability, media resulting in a nil predicted growth rate were filtered out.

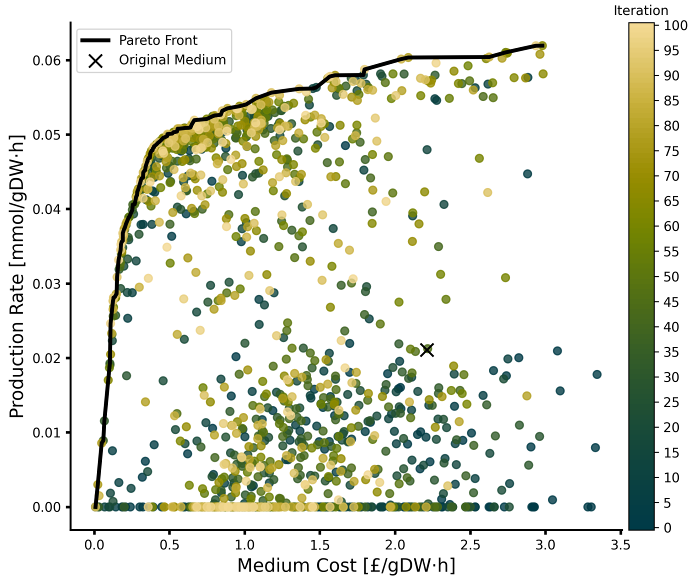

Figure S3: **Double-objective optimization of antibody-producing *E. coli* GEM medium conditions.** The optimization, with the objective to maximize the production rate while minimizing the cost, was run for N=100 iterations, a batch size of 15, and 50 random starting medium compositions.

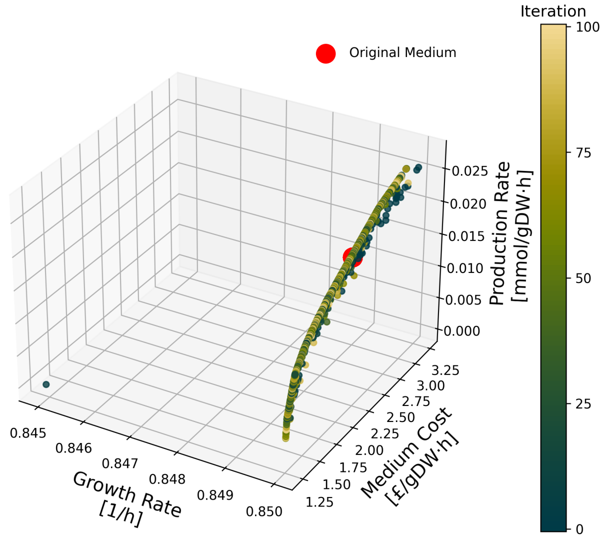

Figure S4: **Triple-objective optimization of antibody production in *E. coli*.** This optimization is analogous to Figure 3, but with fixed M9 medium and only the amino acid components as decision variables.

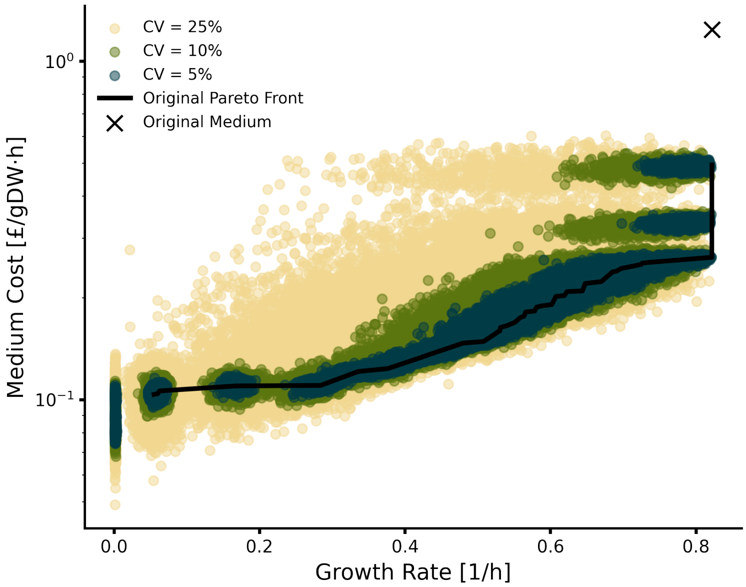

Figure S5: **Sensitivity analysis of Pareto-optimal media compositions in *Escherichia coli* iML1515 model.** Shown is the cost-growth Pareto front (Figure 2A), recomputed with N=4,100 samples (100 initial samples, 200 iterations, and 20 candidates per iteration) for increased resolution, overlaid with the two objectives computed for random compositions. Media compositions were sampled from a multivariate Gaussian distribution with mean μ set to each Pareto-optimal design and increasing standard deviations; the standard deviation was set to 5%, 10%, and 25% of the mean, i.e. $\sigma_i = CV \cdot \mu_i$, with coefficients of variation $CV \in \{0.05, 0.1, 0.25\}$. A total of N=1,000 samples were computed for each Pareto-optimal point and coefficient of variation. Objective values were computed for all samples that lie within the bounds employed for the optimization; samples outside the optimization bounds were filtered out.

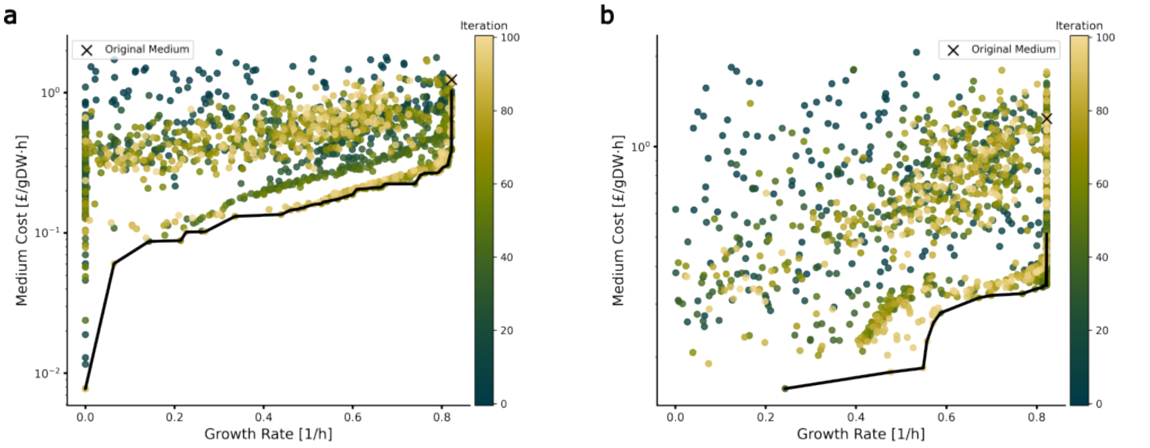

Figure S6: **Equality-constrained optimization of *Escherichia coli* iML1515 model.** We added equality constraints on media components to the double objective optimization from Figure 2. Following the M9 formulation, we employed $[Mg^{2+}]$=$[SO_4^{2-}]$ and $[Cl^-] = 2[Ca^{2+}] + [NH_4^+]$ +($[Na^1]$-2($[PO_4^{3-}]$-$[K^+]$)). (**A**) Cost-growth optimization under equality constraints in media components using standard FBA. (**B**) Cost-growth optimization under equality constraints in media components using parsimonious FBA implemented in COBRApy. For readability, media resulting in a nil predicted growth rate were filtered out.
